# Characteristics of Achalasia Subtypes: Result of a Study in a Tertiary Hospital

**DOI:** 10.22088/cjim.13.1.100

**Published:** 2022

**Authors:** Saba Alvand, Behnaz Aghaee, Zahra Momayez Sanat

**Affiliations:** 1 Liver and Pancreatobiliary Diseases Research Center, Digestive Diseases Research Institute, Tehran University of Medical Sciences, Tehran, Iran; 2 Shariati Hospital, Tehran University of Medical Sciences, Tehran, Iran; 3 Digestive Diseases Research Institute, Tehran University of Medical Sciences, Tehran, Iran

**Keywords:** Achalasia, clinical manifestations, high-resolution manometry, Iran

## Abstract

**Background:**

With the appearance of enhancing high-resolution manometry (HRM), realizing the difference of achalasia symptoms between classified groups by HRM is an outcome of interest in areas with remote access to this device.

**Methods:**

All patients newly diagnosed with achalasia from January 2019 to March 2020 were enrolled in the study. All the patients were diagnosed via HRM after undergoing endoscopy to rule out pseudo-achalasia, and grouped based on the Chicago classification criteria and answered a questionnaire assessing relevant symptoms.

**Results:**

We included 62 patients recently diagnosed with achalasia with a mean age of 53.66 in males (n=30), and 45.4 in females (n=32). Mean time of diagnosis was 24 months. Thirty-seven percent were in type I, 50% in type II, and 13% in type III. Dysphagia and weight loss were higher in type III, while all other relevant symptoms were higher in type II, none of which, however, was statistically significant. Weight loss was reversely associated with duration of symptoms (Spearman correlation= -0.3, P=0.01), and this reverse association was more prominent in females (Spearman correlation= -0.47, P=0.009), type III (Spearman correlation= -0.74, P=0.03), and in the first clinical stages (Spearman correlation= -0.55, P=0.04) in sub analysis.

**Conclusion:**

Type II is the most common type of achalasia in this study. Unlike HRM classification, clinical manifestations alone cannot be used to group patients into different types. However, significant weight loss of the newly diagnosed subjects can become an indicator of on-time diagnosis of the patients.

Achalasia is a rare disease caused by esophagogastric junction obstruction, due to the impaired relaxation of the lower esophageal sphincter along with abnormal peristalsis, presenting with a range of symptoms such as dysphagia, regurgitation, chest pain, heartburn, and weight loss (1, 2). To choose a more efficient treatment approach, the Chicago classification system has grouped the disease into three subtypes based on high-resolution manometry findings, which is the current gold standard of diagnosis (3). Various studies have been conducted to characterize differences in symptoms between the different subgroups. Patel *et al.* mentioned that weight loss was most frequent in type II patients, and the least frequent in type III. They also found that chest pain was most frequently observed among type III patients (4, 5). Several studies have reported that symptoms are experienced differently among men and women, regardless of subtype. This is in accordance with the results found in an Iranian prospective study that reported more frequent chest pain in females (6). Age is another factor affecting the disease’s clinical aspects.

In a comparative study, it was found that elderly patients experience heartburn less frequently than younger patients, even though achalasia is associated with decreased lower esophageal sphincter (LES) pressure (7), this finding was repeated in a similar study conducted in Brazil (8). In order for history taking to replace manometry in patients' follow-up, it is necessary to identify symptoms that are associated with disease severity. Tang *et al.* found that the only indicator found to be associated with disease severity was weight loss which should be applied in type I (9).

The differences among clinical manifestations of the subtypes are controversial. Thus, we aim to report the frequency of achalasia subtypes and the most common symptoms in each subtype, to determine whether the clinical manifestation can guide physician to diagnosis without available HRM, and whether these findings can be used for severity prediction.

## Methods

From January 2019 to March 2020, 62 patients newly diagnosed with achalasia in Shariati Hospital (affiliated to Tehran University of Medical Sciences (TUMS)) were asked to participate in the study. Informed consent was obtained from all patients, and the Ethics Committee of Medical Research at TUMS reviewed and approved this study (Ethics code: IR.TUMS.DDRI.REC.1399.021). All patients had undergone endoscopy after 12 hours of fasting to rule out possible malignancies that can mimic achalasia symptoms (such as dysphagia to liquids or solids and regurgitation), and afterward underwent hyper-resolution manometry (HRM) for an accurate diagnosis.

HRM was performed after 48 hours of liquid diet and 8 hours of fasting in a semi-sitting position using sensors placed 1 cm apart by MMR manometry device made in the Netherlands. Esophageal function was assessed through ten times swallowing of 5 cc water at 15 seconds intervals. During HRM, lower esophageal sphincter (LES) resting pressure, and IRP were measured and recorded. The diagnosis was made based on abnormal body peristalsis and impaired esophagogastric junction relaxation which was defined by an integrated relaxation pressure (IRP) greater than 15 mmHg (10). All the patients underwent an endoscopy to rule out pseudo-achalasia. Further subtype classification was made using the Chicago classification criteria (3). All of the patients answered a questionnaire that primarily targeted persistent symptoms. The questionnaire also assessed relevant symptoms such as dysphagia, regurgitation, reflux, chest pain, nocturnal cough, nocturnal dyspnea, and weight loss. 

The Eckardt symptom score was calculated for each patient according to the frequency of the four symptoms of weight loss, dysphagia, regurgitation, and chest pain (11). Frequency of dysphagia, regurgitation, and chest pain was asked and stands as following: 0= never, 1=occasionally, 2= daily, 3=with every meal, and weight loss was reported in kilogram. The scores were then assigned to the four clinical stages. Scores less than 2 were assigned to stages 0, 2-3 to stages I, 4-6 to stage 2, and scores greater than 6 were assigned to stage 3 (12).

**Statistical Analysis: **We specified the frequencies of variables. Mean and standard deviation were calculated for quantitative variables. The differences between qualitative and quantitative variables across gender, achalasia subgroup, and clinical stage were calculated by cross-tabulation, and ANOVA, respectively. The association between weight loss amount, symptom duration and IRP was explored using the Spearman correlation coefficient. All of the analyses were carried out using SPSS Version 24, and statistical significance was declared if the p-value was less than 0.05.

## Results

**Figure 1:**
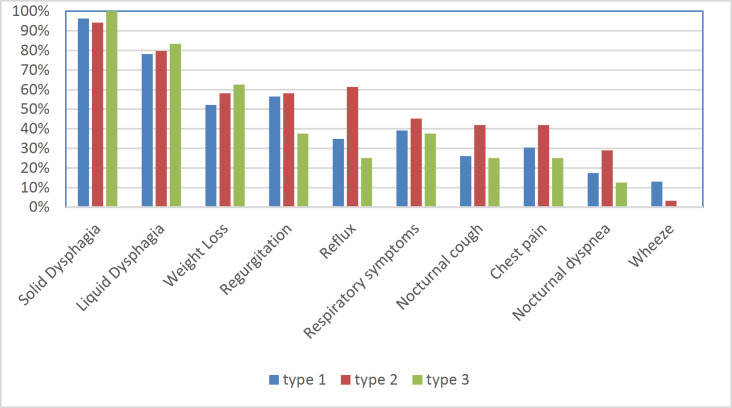
Symptoms among subtypes

**Figure 2:**
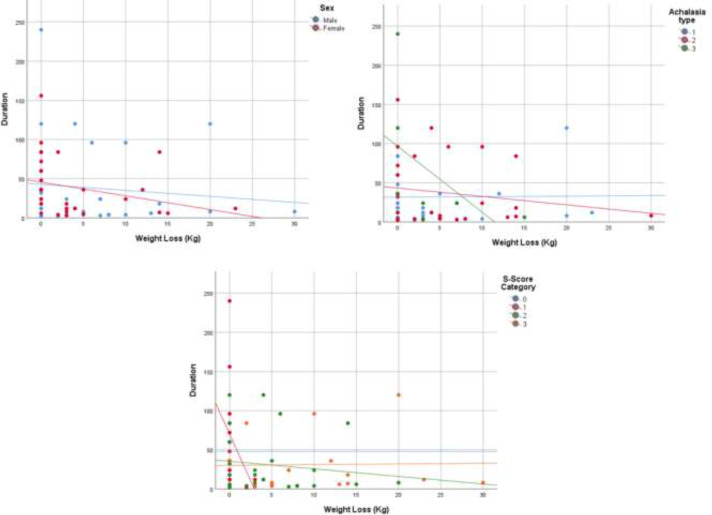
Correlation of weight loss and duration of symptoms among genders, subtypes, and clinical score

Thirty-two participants were males with a mean age of 53.66 (SD=17.66), and 30 participants were females with a mean age of 45.4 (SD=12.83). Half of the participants were in stages II, and 3.2, 22.6, and 24.2 percent were in stages 0, I, and III respectively. Men with achalasia were significantly older in this sample (P=0.04). Table 1 shows the differences between men and women in disease symptoms, clinical score, and IRP. Men and women experienced the same symptoms for similar amounts of time, and both were diagnosed via IRP. There was no difference between the older patients (>60 years old) and younger ones in reporting symptoms and IRP. The chief complaint of 93.5% of patients was dysphagia, and 4.8% most suffered from regurgitation. Table 1 shows the total of all the symptoms in patients. Out of 62 patients with confirmed achalasia by HRM, 37% were classified into type I, 50% into type II, and 13% into type III. The mean age of achalasia was not various among achalasia types (type I: 51.61±15.39, II: 49.55±16.8, and III 44.5±15.31, p=0.56). The main symptoms were as following: dysphagia, weight loss, regurgitation, reflux, chest pain, nocturnal cough, and nocturnal dyspnea. Figure 1 demonstrated the prevalence of the mentioned symptoms among the subtypes I to III. Although chest pain, regurgitation, reflux, and respiratory symptoms (nocturnal cough and dyspnea) were more common in type II than the other two types, the differences were not statistically significant. Amount of weight loss was not associated with IRP (Spearman correlation= 0.04, P=0.72), however, we found evidence of reverse correlation with duration of symptoms (Spearman correlation= -0.3, P=0.01). This association remained high among females (Spearman correlation= -0.47, P=0.009), type III (Spearman correlation= -0.74, P=0.03), and in the first clinical stages (Spearman correlation= -0.55, P=0.04) in contrast to males, type I and II, and other clinical stages that were not independently related with weight loss. For better visualization, the aforementioned correlations are illustrated in figure 2.

**Table 1:** Achalasia characteristics among genders

**Characteristics**	**Total (n=62)**	**Male (n=42)**	**Female (n=40)**	**p-value**
Symptoms duration in month (Mean, range)	24 (7-60)	18 (4-48)	30 (12-60)	0.26
Weight loss (%)	56.5	59.4	53.3	0.41
Weight loss in Kg (Mean, range)	3 (0-7)	4 (0-8)	2 (0-5)	0.35
Chest pain (%)	35.5	31.3	40.0	0.56
Severity (0-3) (Mean)	0.42	0.38	0.47	0.56
Dysphagia (%)	98.4	100.0	96.7	0.48
The severity of dysphagia to solid (0-3) (Mean)	2.53	2.66	2.40	0.22
The severity of dysphagia to liquid (0-3) (Mean)	1.69	1.75	1.63	0.70
Regurgitation (%)	54.8	53.1	56.7	0.49
Severity (0-3) (Mean)	0.92	0.91	0.93	0.91
Reflux (%)	46.8	43.8	50.0	0.40
Nocturnal cough (%)	33.9	31.3	36.7	0.42
Nocturnal dyspnea (%)	22.6	18.8	26.7	0.33
Wheeze (%)	6.5	3.1	10.0	0.28
Eckardt score (Mean ± SD)	5.02±2.18	5.00±2.00	5.00±2.00	0.52
IRP (Mean, range)	22.28 (19.00-28.00)	24.63 (18.00-29.10)	21.06 (19.00-25.60)	0.10

IRP = integrated relaxation pressure

**Table 2:** Achalasia characteristics among subtypes

**Characteristics**	**I ** **(n=23)**	**II ** **(n=31)**	**III ** **(n= 8)**	**P-value**
Symptoms duration in month (Mean, range)	18 (8-48)	18 (5-72)	24 (15-78)	0.77
Weight loss (%)	52.2	58.1	62.5	0.88
Weight loss in Kg (Mean, range)	2 (0-8)	3 (0-8)	3 (0-7)	0.90
Chest pain (%)	30.4	41.9	25.0	0.71
Severity (0-3) (Mean)	0.35	0.48	0.38	0.37
Dysphagia (%)	95.7	100.0	100.0	0.50
The severity of dysphagia to solid (0-3) (Mean)	2.17	2.74	2.75	0.02
The severity of dysphagia to liquid (0-3) (Mean)	1.48	1.77	2.00	0.50
Regurgitation (%)	56.5	58.1	37.5	0.60
Severity (0-3) (Mean)	0.91	0.90	1.00	0.97
Reflux (%)	34.8	61.3	25.0	0.07
Nocturnal cough (%)	26.1	41.9	25.0	0.46
Nocturnal dyspnea (%)	17.4	29.0	12.5	0.58
Wheeze (%)	13.0	3.2	0.0	0.33
Eckardt score (Mean ± SD)	4.52±2.43	5.32±2.12	5.25±1.58	0.39
IRP (Mean, range)	19.10 (16.00-23.70)	25.00 (20.00-29.00)	21.72 (20.62-30.60)	0.006

IRP = integrated relaxation pressure

## Discussion

Achalasia is a rare esophageal motility disorder with an annual incidence of 0.3- 1.63 per 100,000 people that mostly happens in middle-aged groups (13, 14). Barium esophagogram and endoscopy provide clues in the diagnosis of achalasia. In a randomized clinical trial on 245 patients aiming to compare the efficacy of conventional manometry and high-resolution manometry, Fox et al. reached the conclusion that HRM can detect esophageal motility at an earlier stage (15). This, along with other studies has made HRM the gold-standard for the identification of esophageal motility disorders, by which it is possible to measure integrated relaxation pressure, peristalsis, pressurization, and contraction (3, 16). After the discovery of the different types of achalasia, studies have been carried out to determine treatment response among subtypes. In a comparative study in 2008, Pandolfino et al.,found that type II predicts better response in contrast to type III, which was shown to have the poorest outcome for treatment (1, 10). This result was in accordance with the report of the two later meta-analyses in 1575 and 727 patients (17, 18). 

This progress in diagnosis and discovering motility differences raised the question of whether the various subtypes suffer from different symptoms. To answer this question, we designed a study with acceptable numbers of patients newly diagnosed by HRM. Out of the 62 patients enrolled in the study, the most common type according to Chicago classification criteria was type II (50%), following by type I and type III with 37%, and 12.9%, respectively. Our frequency was compatible with the 2020 report American College of Gastroenterology, in which type II was the most common type consisting 50-70% of all achalasia patients, followed by type I and type III (19).

**Symptoms:** Dysphagia was the most commonly reported symptom in all subtypes, and nocturnal dyspnea was the least experienced symptom. In a study of 146 patients recently diagnosed with achalasia, dysphagia was the highest reported symptom (94%) followed by regurgitation, while chest pain and weight loss were the least common with 41%, and 35% reporting these symptoms, respectively (20). In 2011, in a study on 110 patients aiming to identify respiratory symptoms in achalasia, 37% of patients reported cough, 10% reported dyspnea, and wheeze was found in 17% of patients (21). In a review aiming to measure the prevalence of symptoms, dysphagia was the most probable symptom in 82-100% of patients, following by regurgitation, weight loss, chest pain, and respiratory symptoms (4). We reported a similar pattern of symptoms, but men reported weight loss more commonly than regurgitation, unlike females. This can be due to the older age of male subjects. However, this difference was not statistically significant. Many studies focused on the differences in experienced symptoms between genders and age groups. In a study among 101 patients over 18 years old, chest pain was significantly more common among females and young patients compared to males and elderly patients, and the severity of it decreased with age in affected subjects (22). 

In a study by Mikaieli et al., chest pain was investigated in both genders before and after treatment, resulting in a significant decrease after treatment in both sexes. However, it was still higher in females than males after treatment, and patients younger than 56 years old were more likely to report it (6). Other studies reported similar results (7, 23). For a better assessment of age and achalasia, Ribeiro et al.designed a study on elderly patients with and without achalasia and reported that older patients have lower IRP than younger patients with achalasia, and elderly patients with dysphagia are more likely to have esophageal motility disorders than youngsters with similar complaints (24). We did not include all the patients with the complaint of dysphagia in this study, therefore we cannot discuss the specificity of the self-report of dysphagia in various age ranges. However, in our sample, there was no difference between IRP among age groups, in accordance with Robson's study in 2003. Although age affects the peristalsis and LES pressure of the esophageal body,(8, 25, 26) Robson et al. stated, it does not necessarily raise the risk of achalasia in the elderly (27). 

**Difference in subtypes: **Type II patients experienced all symptoms more severely and more frequently than the other two groups, except for weight loss, which was more frequent in type III. Type I and type III were quite similar in all symptoms except for regurgitation. Despite the mentioned comparisons, reported symptoms were not significantly different among the subtypes in this study. Whereas in Patel's study, chest pain was more common in type III patients, and weight loss in type I. This can be due to the various duration of the disease among achalasia subtypes, whereas in our study all the three subtypes had similar duration of symptoms, making it more accurate in symptoms comparison (28). In a study in 2018, weight loss was reported more frequently in type II, and in subjects without weight loss, the other symptoms of achalasia existed for a longer period (12 months vs. 24 months) (28). In our study, weight loss was reversely correlated with the duration of other symptoms, especially in females and in type III patients. While Hassanzadeh et al. found weight loss to be the fifth most common symptom, it was the second most likely symptom in our sample. This difference can be due to the earlier detection of achalasia in our study; The average time of detection was 2 years (7month to 5 years), half of that of Hassanzadeh's study (4.9 years), and Eckardt’s investigations (11, 23, 29). The awareness of new clinicians and increased accessibility to HRM in the country can be the main reasons for this dramatic decline. The reduction of all the symptoms except for achalasia in our study in proportion to Hassanzadeh’s is a further evidence for the earlier referral of patients with dysphagia to gastroenterologists. One of the strengths of our research is choosing a tertiary referral center for the study. Thus, the population of diagnoses can be representative of the region. Inability to group patients according to the exact pathology like Chagas disease was the main limitation of the study that can be a bias to the symptoms of the different subgroups. Also, the sample size could have been larger with a wider time spectrum to obtain more accurate results.

In conclusion, Achalasia is a rare disease, resulting in the misdiagnosis of patients and increased expenses due to mistreatment. By regular assessment of diagnosed patients, the pattern of physicians’ awareness can be estimated. Weight loss is more common in earlier stages, and its accompaniment with dysphagia can point towards achalasia. High reports of weight loss among recently referred patients can be an indicator of on-time diagnosis.

## Funding:

The authors received no financial support for the research, authorship, and/or publication of this article.

## Conflict of interest:

The authors declare that there is no conflict of interest.
